# KLF4 downregulates hTERT expression and telomerase activity to inhibit lung carcinoma growth

**DOI:** 10.18632/oncotarget.9141

**Published:** 2016-05-02

**Authors:** Wenxian Hu, Yunlu Jia, Xiangsheng Xiao, Kezhen Lv, Yongxia Chen, Linbo Wang, Xiao Luo, Tianze Liu, Wenbin Li, Yixin Li, Changlin Zhang, Zhenglong Yu, Wenlin Huang, Bing Sun, Wu-guo Deng

**Affiliations:** ^1^ Department of Surgical Oncology, Sir Run Run Shaw Hospital, College of Medicine, Zhejiang University, Hangzhou, China; ^2^ Sun Yat-Sen University Cancer Center, State Key Laboratory of Oncology in South China, Collaborative Innovation Center of Cancer Medicine, Guangzhou, China; ^3^ Department of Breast Center, The First Affiliated Hospital, College of Medicine, Zhejiang University, Hangzhou, China; ^4^ Department of Thoracic Surgery, The First Affiliated Hospital of Dalian Medical University, Dalian, China; ^5^ State Key Laboratory of Targeted Drug for Tumors of Guangdong Province, Guangzhou Double Bioproduct Inc., Guangzhou, China

**Keywords:** KLF4, hTERT, MAPK, lung cancer, cancer prognosis

## Abstract

Krüppel-like factor 4 (KLF4) is a transcription factor that contributes to diverse cellular processes and serves as a tumor suppressor or oncogene in various cancers. Previously, we have reported on the tumor suppressive function of KLF4 in lung cancer; however, its precise regulatory mechanism remains elusive. In this study, we found that KLF4 negatively regulated hTERT expression and telomerase activity in lung cancer cell lines and a mouse model. In addition, the KLF4 and hTERT expression levels were significantly related to the clinicopathological features of lung cancer patients. Promoter reporter analyses revealed the decreased hTERT promoter activity in cells infected with Ad-KLF4, and chromatin immunoprecipitation analysis demonstrated that endogenous KLF4 directly bound to the promoter region of hTERT. Furthermore, the MAPK signaling pathway was revealed to be involved in the KLF4/hTERT modulation pathway. Forced expression of KLF4 profoundly attenuated lung cell proliferation and cancer formation in a murine model. Moreover, hTERT overexpression can partially rescue the KLF4-mediated suppressive effect in lung cancer cells. Taken together, these results demonstrate that KLF4 suppresses lung cancer growth by inhibiting hTERT and MAPK signaling. Additionally, the KLF4/hTERT/MAPK pathway is a potential new therapeutic target for human lung cancer.

## INTRODUCTION

Despite advances in early diagnosis and combined therapy, lung cancer remains a major public health problem worldwide. In 2014, approximately 224,000 new cases of lung cancer were diagnosed, and lung cancer accounted for nearly one-quarter of all cancer deaths in the United States [1]. General therapy for lung cancer consists of surgical tumor resection, multimodal chemotherapy, radiation therapy, or a combination of these regimens. However, the lung cancer prognosis remains poor, and more effective therapies are urgently needed. The aggressive nature of lung cancer is associated with various molecular abnormalities, including the loss of tumor suppressor genes, activation of oncogenes and somatic mutations of growth factor receptors [2]. Thus, a better understanding and improved identification of novel factors involved in lung cancer development and progression are essential for defining tumor classification, improving diagnostic accuracy, predicting patient outcomes and providing more effective treatment strategies.

Krüppel-like factor 4 (KLF4; also called gut-enriched Krüppel-like factor or GKLF) was initially identified in postmitotic, terminally differentiated epithelial cells of the skin and the gastrointestinal tract [3, 4]. KLF4 is a zinc finger transcriptional factor involved in regulating numerous physiological processes, including proliferation, apoptosis, differentiation and tumor formation [5–7]. Moreover, as a core member of the pluripotency transcriptional network, KLF4 has gained recognition in the reprogramming of somatic cells into a stem cell-like state [8, 9]. In addition, KLF4 exhibits anti-apoptotic activity upon DNA damage via the cell cycle checkpoint functions of p53 and by blocking cell cycle progression at the G1/S boundary [10–11]. There is mounting evidence that KLF4 functions as an oncogene or tumor suppressor depending on the tumor type. Our previous studies have shown that the expression of KLF4 is dramatically reduced in primary lung cancer tissue and that it functions as a tumor suppressor in primary lung cancer [12]. KLF4 itself has been identified as a tumor suppressor in some tumor types, such as lung cancer, gastric cancer, colorectal cancers, urothelial cancer and cervical cancer [12–17]. Conversely, an oncogenic function of KLF4 has been observed in primary breast ductal carcinoma and oral and skin squamous carcinoma. In primary breast cancer, KLF4 is required for maintaining stem cell-like features after promoting cellular invasion and migration [18–20]. Thus, KLF4 plays an important role in tumor development and progression, while the molecular basis of this process requires further elucidation. Despite the tumor-inhibitory effects of KLF4 in primary lung cancer, few investigations have focused on its identified transcriptional downstream targets

Telomerase is an RNA-dependent DNA polymerase ribonucleoprotein enzyme at the chromosome ends that synthesizes repeat DNA sequences to maintain genome stability [21]. Human telomerase ribonucleoprotein enzyme consists of two core components: the template-containing RNA component (human telomerase RNA, hTR) and the catalytic protein subunit (human telomerase reverse transcriptase, hTERT) [22–23]. In most normal human somatic cells, telomerase shortening results in programmed growth arrest or replicative senescence, while telomerase activation has been implicated in immortalized cells and may therefore increase the cancer risk while maintaining the growth potential [24]. Telomerase is reactivated in most advanced cancers, but it is present at a very low or almost undetectable level in normal cells [25–26]. Different telomerase expression levels are clearly observed between cancer cells and normal human cells, enabling the development of targeted telomerase cancer therapeutic approaches [27–28]. hTERT, which is also known as the reverse transcriptase component of telomerase, is the rate-limiting indicator of telomerase activity, and it plays an important role in cancer-related telomerase function [29]. In addition, hTERT is an identical target of Wnt/β-catenin signaling in cancer cells, while KLF4 directly interacts with Wnt/β-catenin signaling to suppress Wnt signaling and inhibit tumor growth [30–32]. Thus, we hypothesize that KLF4 may play an important role in regulating telomerase activation and hTERT expression in lung carcinoma.

In this study, we aimed to examine the role of KLF4 and its putative target, hTERT, in lung cancer development and progression, which was followed by elucidating the underlying molecular mechanisms and clinical significance. This report provides the first comprehensive analysis of the KLF4/hTERT/MAPK pathway in parallel studies involving human lung cancer tissues, a panel of human lung cancer cells and animal models. Recognition of these concepts will increasingly promote the development of new strategies for treating human cancer.

## RESULTS

### KLF4 was correlated with hTERT expression in lung cancer, and their expression was associated with patient survival

Previously, we showed that KLF4 functions as a tumor suppressor in primary lung cancer. To identify the mechanism by which KLF4 regulates lung cancer growth, we searched for potential targets of KLF4 that are implicated in tumor development and progression. The current knowledge indicates that hTERT might act as a lung cancer gene. A susceptibility locus at 5p15.33 containing hTERT was significantly associated with the lung cancer risk [33, 34]. A longer telomere length predicted a higher risk of lung cancer in patients [35]. Taken together, these findings indicated that there might be crosstalk between hTERT and KLF4 in lung carcinoma.

**Figure 1:**
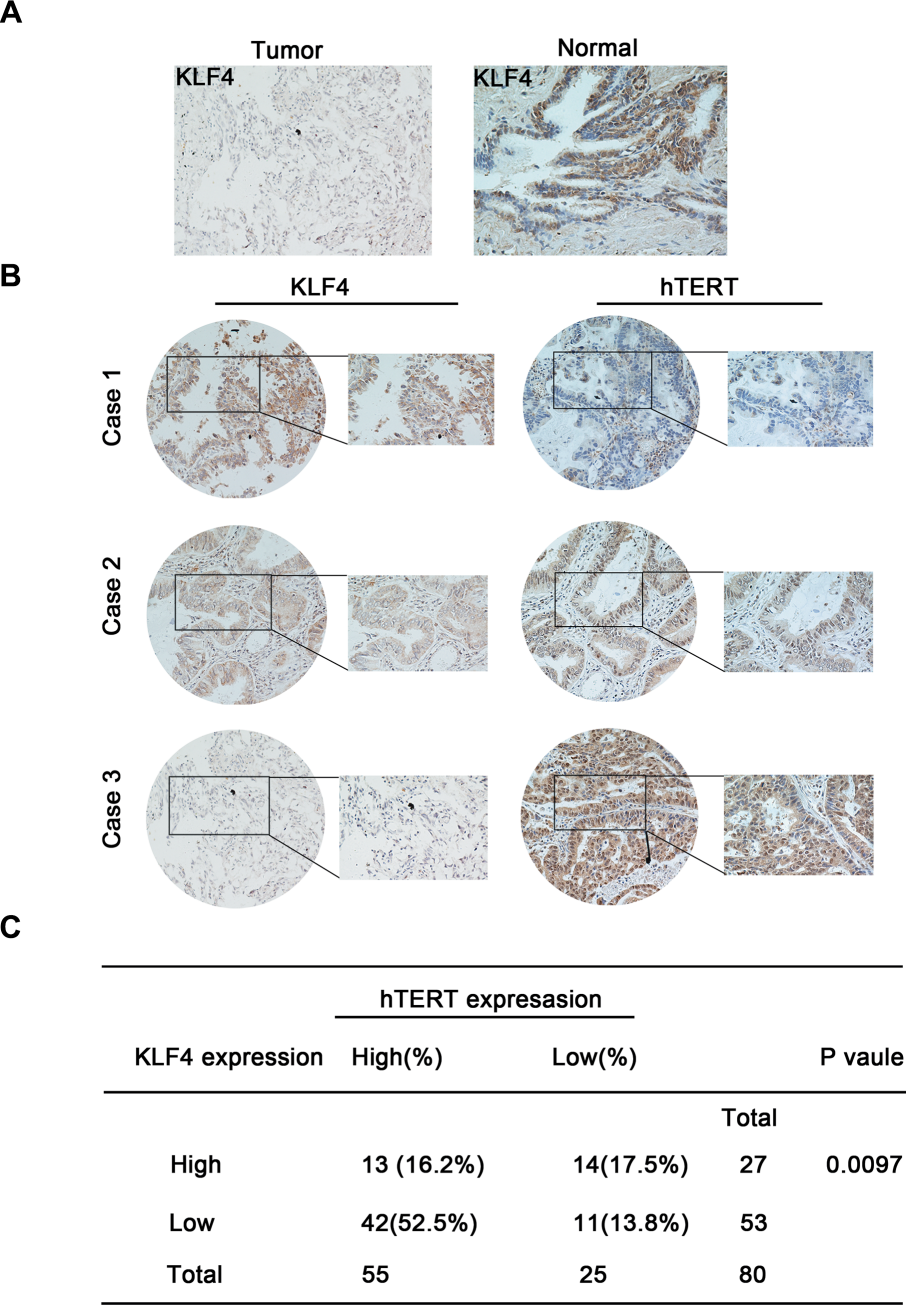
KLF4 and hTERT expression in lung tumor specimens and their correlation with patient outcomes (**A**) Lower KLF4 levels were observed in lung tumor tissues compared with normal adjacent tissues. KLF4 expression is stained as brown. (**B**) Shown are representative images of KLF4 and hTERT staining using immunohistochemical staining for three different cases. (**C**) Correlation between the KLF4 and hTERT expression levels in lung carcinoma tissues obtained from 80 patients.

Next, we systematically assayed the KLF4 and hTERT protein levels using immunohistochemical staining of tissue microarrays, including lung cancer tissues and their comparable normal counterparts. Cases were staged according to the current tumor–node–metastasis (TNM) classification of malignant tumors (International Union Against Cancer, UICC, 7th edition, 2009). We found lower levels of KLF4 in tumor tissues compared with normal adjacent tissues. (Figure 1A). Representative images of three cases are shown in Figure 1B. The correlation between the expression of KLF4 and hTERT in lung cancer tissues was showed in Figure 1C. Among 80 patients' tumor tissue samples tested, about 42 patients showed high expression of hTERT and low expression of KLF4 which accounts for 52.5% of all samples. We also analyzed KLF4 and its relevant factors in lung cancer using a data portal (cBioPortal for Cancer Genomics). The results showed that a crosstalk between TERT and KLF4 existed in lung carcinoma. The cBioPortal for Cancer Genomics (http://cbioportal.org) provides exploration, visualization and analyzing of large-scale cancer genomics data. In silico analysis using the cBioPortal for Cancer Genomics showed that the protein KLF4 is deregulated in lung cancer whereas TERT steadily presented amplification (Supplementary Figure S1A). A mutual exclusive relationship between KLF4 and TERT expression levels was revealed in Lung Adenocarcinoma (TCGA, Nature 2014, 230 patients/230 samples) (Supplementary Figure S1B).

**Figure 2:**
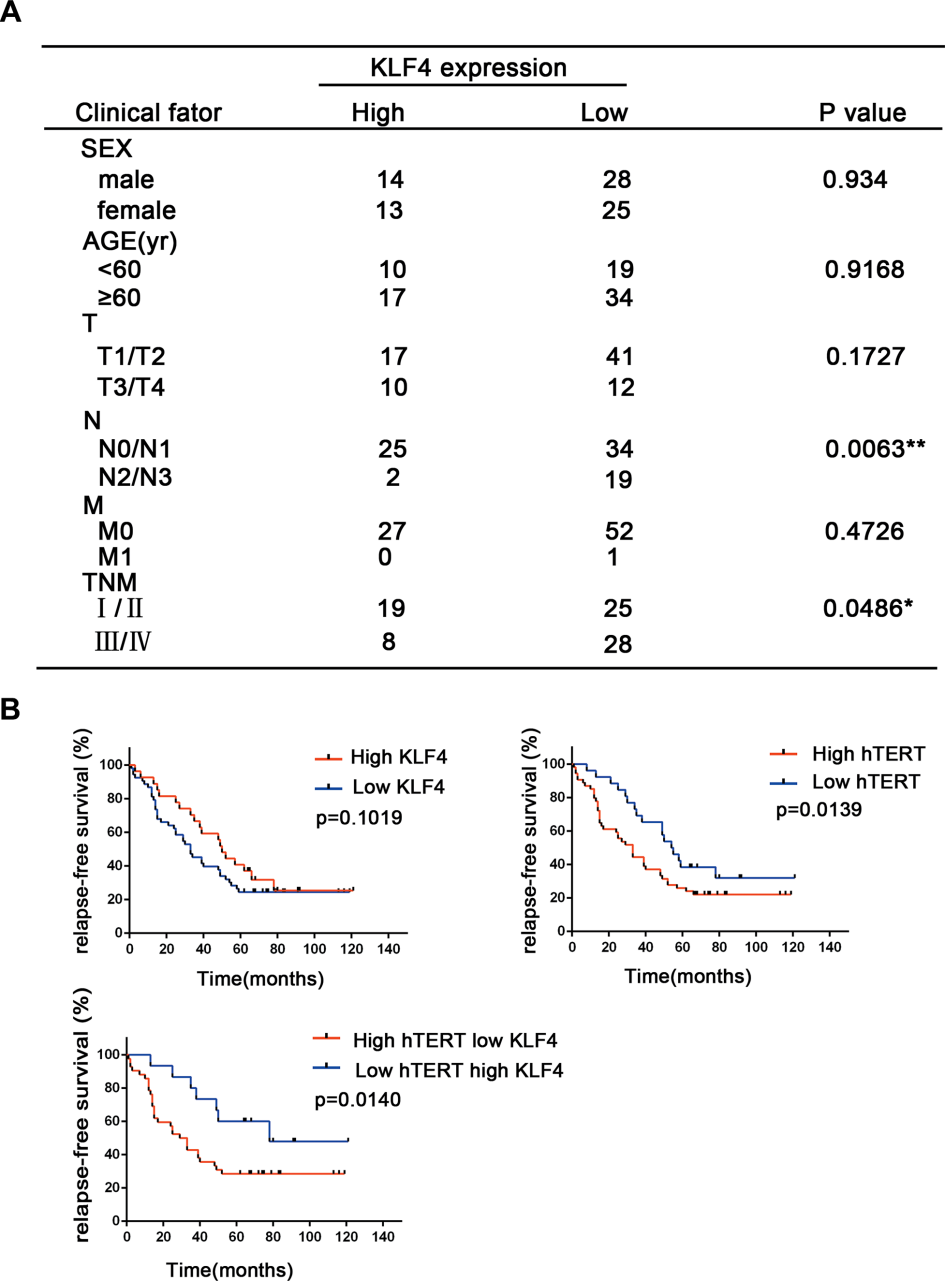
The correlation between the KLF4 and hTERT expression levels with lung cancer patient outcomes (**A**) Correlation analyses of KLF4 protein expression associated with clinicopathological variables in 80 lung carcinoma patients. (**B**) Survival analysis of the relapse-free survival in lung cancer patients with high or low KLF4 and hTERT expression.

The patients' clinical parameters are presented in Figure 2A. There were no significant differences in KLF4 expression between the two KLF4 categories with regard to gender, age and distant metastasis. However, late-stage lung cancer patients showed a marked loss of KLF4 expression (*p* = 0.0486) and a significant association in the distribution of patients according to metastatic lymph nodes (*p* = 0.0063), suggesting that the loss of KLF4 expression might contribute to lung cancer progression. In the survival analysis of patients, Kaplan-Meier analyses supported the observation that decreased KLF4 and hTERT overexpression levels were associated with inferior survival duration (*p* = 0.014). Moreover, the survival time for patients with a high hTERT level was significantly shorter than for patients with lower levels (*p* = 0.0139), while KLF4 expression alone could not predict the prognosis (*p* = 0.109) (Figure 2B). Overall, these results indicated the prognostic implications of KLF4 and hTERT levels in lung tumor.

### KLF4 negatively regulated hTERT expression in lung cancer cell lines

**Figure 3:**
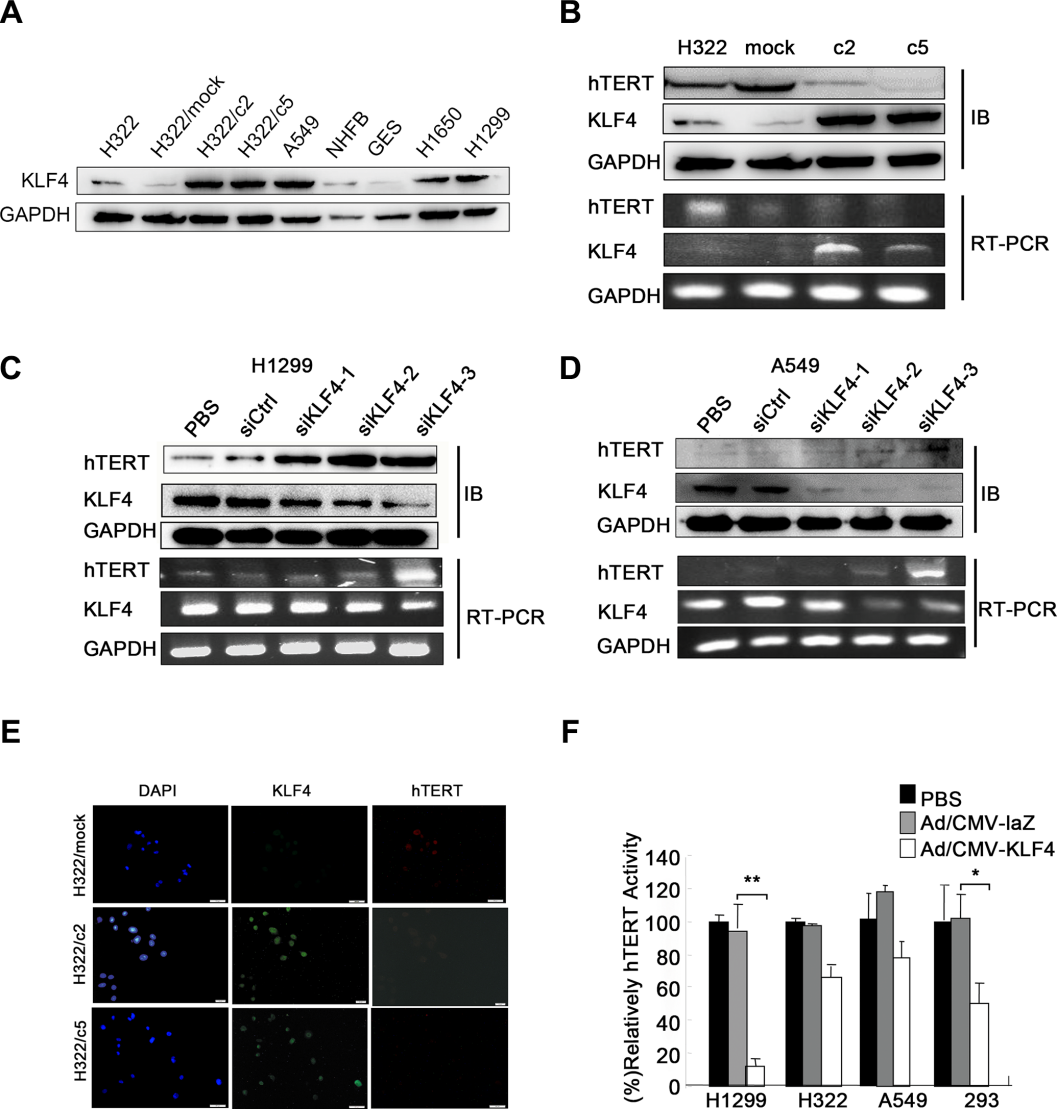
KLF4 negatively regulated hTERT expression in lung cancer cell lines (**A**) KLF4 expression in various lung cancer cell lines was analyzed using Western blot analyses. GAPDH was used as a control. (**B**) Down-regulation of hTERT mRNA and protein expression levels induced by KLF4 overexpression. KLF4 and hTERT expression levels were measured in H322, H322/mock, H322/c2 and H322/c5 cells using Western blot (up) and RT-PCR (down) analyses. GAPDH was used as an internal control. (**C–D**) KLF4 knockdown resulted in hTERT overexpression at the mRNA and protein levels. H1299 and A549 cells were transfected with control nontargeting (Ctrl) or KLF4 siRNA; KLF4 and hTERT expression levels were detected using Western blot (up) and RT-PCR (down) analyses. GAPDH was used as an internal control. (**E**) H322/mock, H322/c2 and H322/c5 cells were stained for KLF4 (green staining) and hTERT (red staining) and analyzed using fluorescence microscopy. Nuclei were stained with DAPI (blue staining). Scale bar, 50 μm. (**F**) Telomerase activity was measured in H1299, H322, A549 and 293 cells. Cells infected with Ad/KLF4 showed significantly decreased hTERT activity. (*P < 0.05 **P < 0.01).

To examine the role of KLF4 in regulating hTERT expression in lung cancer cells, we first detected KLF4 expression in various lung cancer cells at the protein level (Figure 3A). KLF4-stable overexpressing clones of H322 cells (c2 and c5) caused downregulation of hTERT expression (Figure 3B). In contrast, KLF4 knockdown in H1299 and A549 cells resulted in significantly increased hTERT levels at both the protein and mRNA levels (Figure 3C, 3D). As shown in the figures, of the three specific KLF4 siRNA, H1299 cells and A549 cells infected with siKLF4-2 and siKLF4-3 showed a marked suppression of KLF4 levels. Consistent with these results, immunofluorescence staining of H322/mock cells revealed elevated hTERT expression compared to in H322/c2 and H322/c5 cells (Figure 3E). In addition, cancer cells transfected with Ad-KLF4 had profoundly decreased hTERT activity, especially in H1299 cells (Figure 3F). These findings support an inverse association between KLF4 and hTERT in lung cancer cell lines.

### KLF4 suppressed lung cancer cell growth and decreased hTERT expression level via MAPK signaling pathway

**Figure 4:**
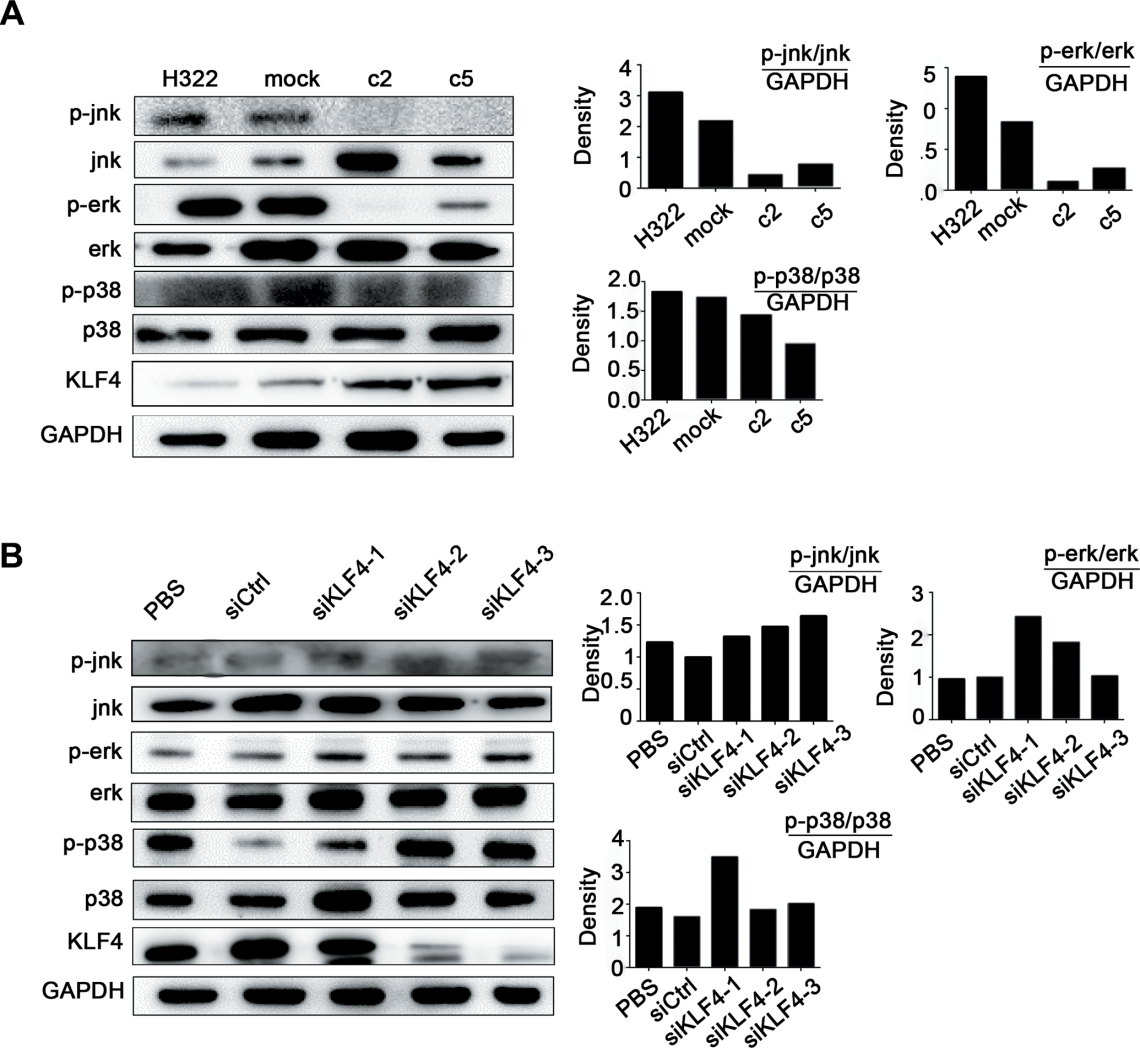
KLF4 suppressed lung cancer cell growth and hTERT expression via the MAPK signaling pathway (**A**) Western blot analysis indicated decreased levels of the phosphorylation of jnk, erk, p38 in KLF4 stable-overexpression clones (H322/c2 and H322/c5). The quantitative measurement is shown next to the Western blot. (**B**) Western blot analysis indicated that H1299 cells transfected with KLF4 siRNA presented increased levels of jnk, erk and p38 phosphorylation, in contrast with cells transfected with siCtrl. The quantitative measurement is shown next to the Western blot.

To further understand the mechanisms by which KLF4 regulates hTERT expression, we examined putative upstream regulators and downstream targets. The MAPK signaling pathway is not only responsible for different cellular processes, such as proliferation, differentiation and development [36], but also involved regulating telomerase activity during diverse cellular processes [37–39]. To determine whether KLF4 could suppress cancer cell growth and hTERT expression via this pathway in our model, the expression levels of the MARK pathway-related molecules were examined. Western blot analysis showed a significant decrease in p-jnk, p-erk, and p-p38 in KLF4-overexpressing cell clones (H322/c2, H322/c5) compared with H322/mock cells (Figure 4A). Conversely, the loss of KLF4 exerted the opposite effects (Figure 4B). These results suggested that the MAPK signaling pathway might be involved in the process by which KLF4 regulates hTERT.

### KLF4 directly bound to a specific region of the hTERT promoter

**Figure 5:**
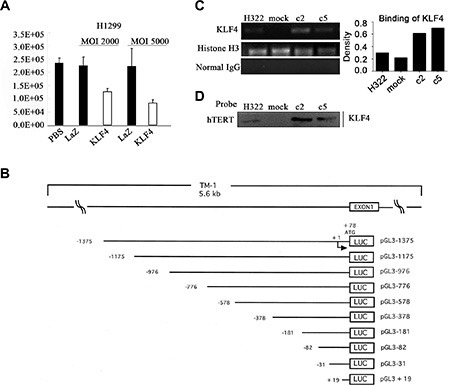
KLF4 directly bound to the promoter region of hTERT (**A**) Luciferase reporter assay revealed the effect of KLF4 on the hTERT promoter activity. H322, A549, H1299 and 293 cells were transfected with a luciferase reporter construct harboring the promoter region of hTERT with Ad-KLF4. Error bars indicate the SEM. (**B**) The structures of hTERT promoter-luciferase constructs. A schematic representation of reporter plasmids. 1.4 kb DNA and 5'-truncated fragments of hTERT promoter upstream of the initiating ATG were inserted into luciferase (LUC) reporter vector pGL3-Basic in sense orientation. Arrow, the tran-scription start site. Numbers, the number of bases upstream (–) and downstream (+) of the transcrip-tion start site. The name of each reporter construct was assigned according to the 5'-end nucleotide numbers of inserted promoter sequences, upstream (–) or downstream (+) of the transcription start site. (**C**) Chromatin immunoprecipitation (ChIP) analysis of KLF4 binding to the target region on the hTERT promoter. Chromatin was obtained from H322, H322/mock, H322/c2 and H322/c5 cells, and a specific KLF4 antibody was used to detect the binding of KLF4 to the hTERT promoter in these cells. Normal rabbit IgG was used as a negative control and histone H3 was used as a positive control. The quantitative measurement is shown. (**D**) A streptavidin-agarose pulldown assay was performed with the hTERT promoter as a probe. The pulled-down protein complex was revealed using Western blot analysis with an anti-KLF4 antibody.

To further elucidate the mechanisms of the KLF4-mediated modulation of hTERT, the Ad-KLF4 or control plasmid Ad-LacZ was transfected into H1299 cells combined with the hTERT promoter-luciferase reporter plasmid. KLF4 overexpression significantly inhibited the hTERT reporter activity, indicating that increased KLF4 resulted in decreased recruitment to the hTERT promoter (Figure 5A). The structures of hTERT promoter-luciferase constructs were shown in Figure 5B, which were cloned previously. We assayed the binding of KLF4 to the hTERT promoter in H322, H322/mock, H322/c2 and H322/c5 cells. The ChIP assay was performed using an anti-KLF4 antibody. The anti-histone H3 antibody was used as a positive control and normal rabbit-IgG was used as a negative control. Samples treated with the anti-KLF4 antibody had a significant enrichment of the hTERT promoter sequence, indicating that KLF4 directly bound to regions of the hTERT promoter (Figure 5C). These results were further confirmed using the streptavidin-agarose pulldown assay with a specific hTERT promoter probe. Cells overexpressing KLF4 had significantly increased binding to the hTERT promoter (Figure 5D). These findings demonstrated that KLF4 suppressed hTERT by directly binding to the promoter region of hTERT.

### Exogenous KLF4 expression restrained tumor cell growth and overexpression of hTERT rescued KLF4-mediated inhibition of cell proliferation

**Figure 6:**
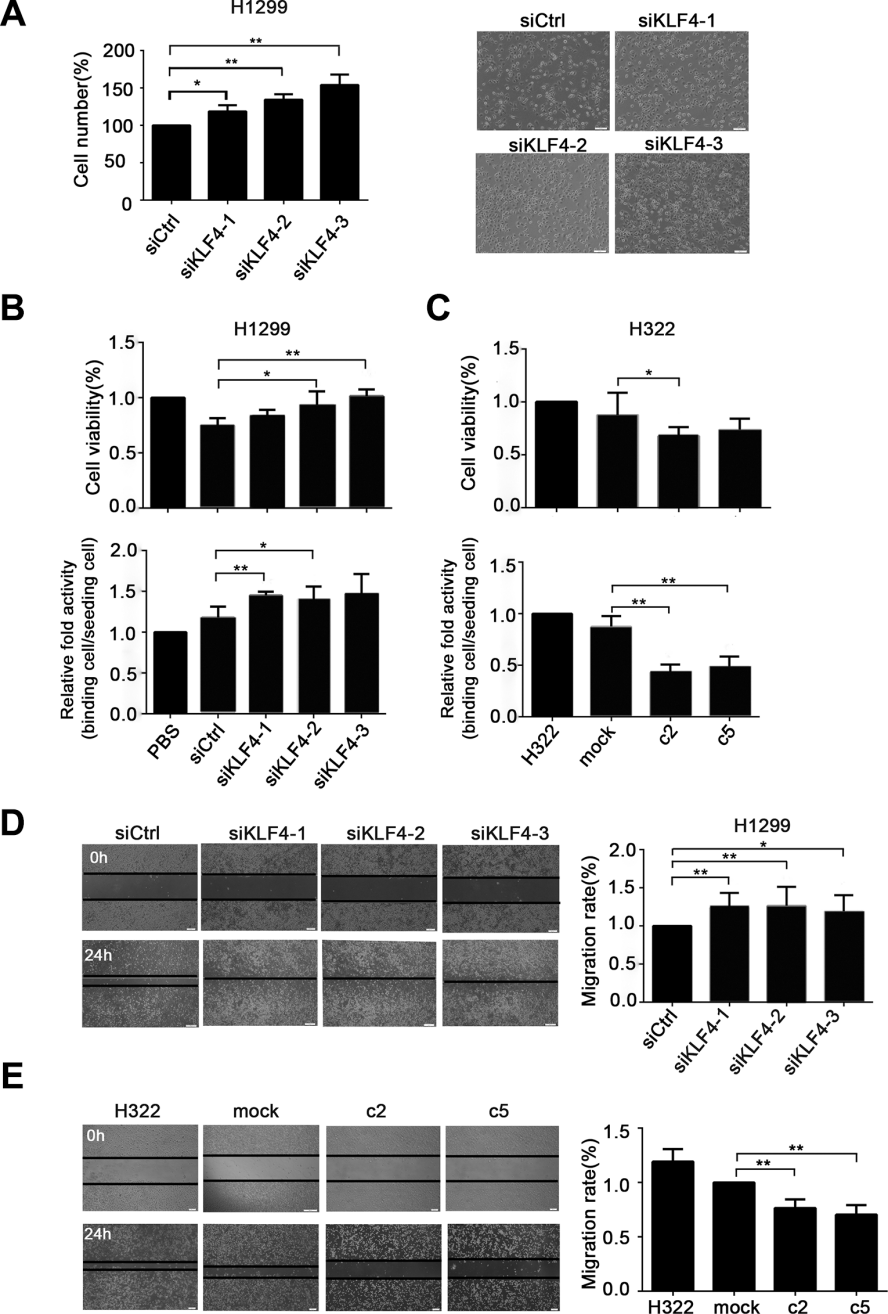
KLF4 knockdown promoted lung cancer cell growth, migration and adhesion ability *in vitro* (**A**) Pronounced changes in the cell counts in H1299 cells infected with KLF4 siRNA compared with control nontargeting (Ctrl). Data are presented as the mean ± SD. Bar graph, quantification of the cell numbers. *P* < 0.05 in a comparison between cells transfected with KLF4 siRNA and siCtrl. (**B**) KLF4 knockdown cells demonstrated an increased cell viability and adhesion capacity. H1299 cells were transfected with KLF4 siRNA or siCtrl. Data are presented as the mean ± SD. Bar graph, quantification of the relative cell viability or relative binding cells. *P* < 0.05 in comparison between cells transfected with KLF4 siRNA and siCtrl. (**C**) Overexpression of KLF4 resulted in decreased cell viability and adhesion capacity. Data are presented as the mean ± SD. Bar graph, quantification of the relative cell viability or relative binding cells. *P* < 0.05 in a comparison between H322/mock and H322/c2 or H322/c5 cells. (**D**) KLF4 knockdown promoted the migration ability of H1299 cells, as measured using the scratch assay. Data are presented as the mean ± SD. Bar graph, quantification of the migration distance. *P* < 0.05 in a comparison between cells transfected with KLF4 siRNA and siCtrl. (**E**) KLF4 expression levels affected the migration ability of H322 cells. The scratch assay showed that overexpression of KLF4 in c2 and c5 cells had lower migration ability than mock cells. *P* < 0.05 in a comparison between mock cells and c2 or c5.

To investigate the biological activities of KLF4 in lung cancer cells, we first knocked down KLF4 expression in H1299 cells, which caused significantly increased cell proliferation compared with the control siRNA (siCtrl) treated cells (Figure 6A). KLF4 slicing in H1299 cells exhibited significantly increased cell proliferation and adhesion ability compared with nontargeting siRNA (Figure 6B). Conversely, compared to control cells (H322/mock), KLF4-overexpressing cells (H322/c2 and H322/c5) had a much lower proliferation ability and adhesion capacity, as measured using the MTS (3-(4,5-dimethylthiazol-2-yl)-5-(3-carboxymethoxyphenyl)-2-(4-sulfophenyl)-2H-tetrazolium, inner salt) and cell adhesion assays (Figure 6C). Moreover, loss of KLF4 consistently increased the cell migration ability in a wound closure assay (Figure 6D). Conversely, KLF4-overexpressing cells (H322/c2 and H322/c5) had a lower migration ability compared to mock cells (Figure 6E). Taken together, these findings suggested that KLF4 suppressed cancer cell growth, migration and adhesion.

**Figure 7:**
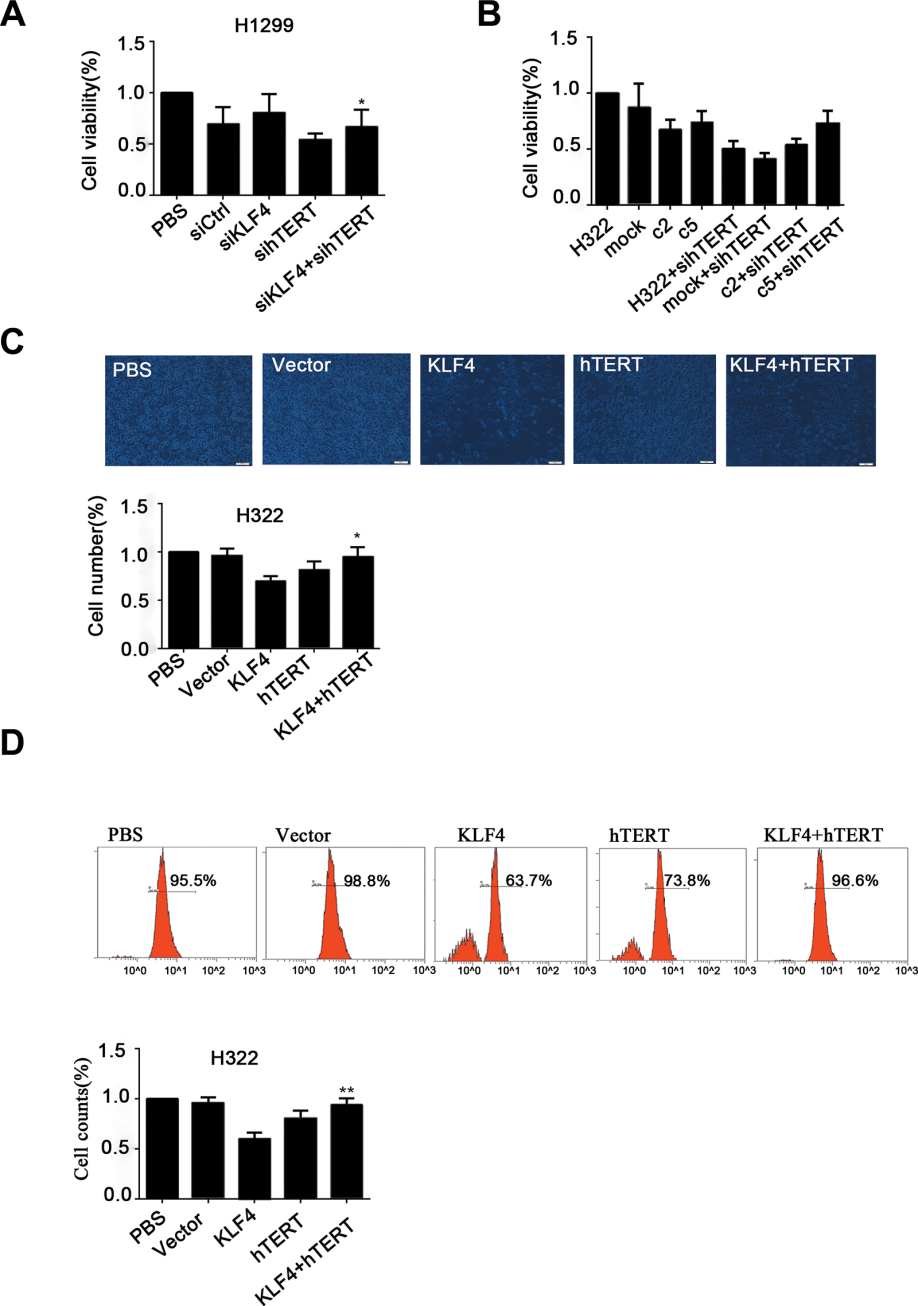
hTERT up-regulation rescued KLF4-mediated inhibition of cancer cell growth (**A**) H1299 cells transfected with hTERT siRNA and KLF4 siRNA can suppress KLF4 siRNA-mediated enhancement of cancer cell viability as measured using the MTS assay. Data are presented as the mean ± SD. Bar graph, quantification of the relative cell viability. *P* < 0.05 in a comparison between cells transfected with KLF4 siRNA and cells transfected with KLF4 siRNA and hTERT siRNA. (**B**) KLF4-overexpressing cells (H322/c2 and H322/c5) infected with hTERT siRNA demonstrated a significantly decreased proliferation rate. Data are presented as the mean ± SD. Bar graph, quantification of relative cell viability. (**C**) Robust changes in the cell counts in H322 cells transfected with Ad-KLF4 compared with cells transfected with Ad-KLF4 plus Ad-hTERT. Statistical graphs of the cell-counting assay are shown. Data are presented as the mean ± SD. Bar graph, quantification of relative cell number. *P* < 0.05 in a comparison between cells transfected with Ad-KLF4 and cells transfected with Ad-KLF4 plus Ad-hTERT (**D**) The proliferation ability was examined in H322 cells transfected with Ad-KLF4 or Ad-hTERT. Statistical graphs of the EdU proliferation assay are shown. Data are presented as the mean ± SD. Bar graph, quantification of relative cell counts. *P* < 0.05 in a comparison between cells transfected with Ad-KLF4 and cells transfected with Ad-KLF4 plus Ad-hTERT.

Given that KLF4 has been shown to suppress hTERT expression and cell growth, we examined whether hTERT could contribute to KLF4-mediated inhibition of cancer cell proliferation. The MTS assay was performed in H1299 cells that were infected with KLF4 siRNA or hTERT siRNA. A positive effect of KLF4 knockdown on cell proliferation could be partially ameliorated by hTERT slicing (Figure 7A). Compared with H322/mock cell, KLF4 overexpression in c2 and c5 resulted in decreased cell viability. Moreover, knockdown of hTERT could significantly suppress the proliferation of these cells. (Figure 7B). These findings were confirmed using two additional assays for proliferation, cell-counting and EdU proliferation assays. Overexpression of hTERT in H322/c2 and H322/c5 cells could significantly compensate for the KLF4-mediated inhibition of cell proliferation (Figure 7C, 7D). Taken together, these data demonstrated that enforced restoration of hTERT expression rescued KLF4-mediated cell growth depression *in vitro*.

### KLF4 suppressed tumor formation and hTERT expression *in vivo*

**Figure 8:**
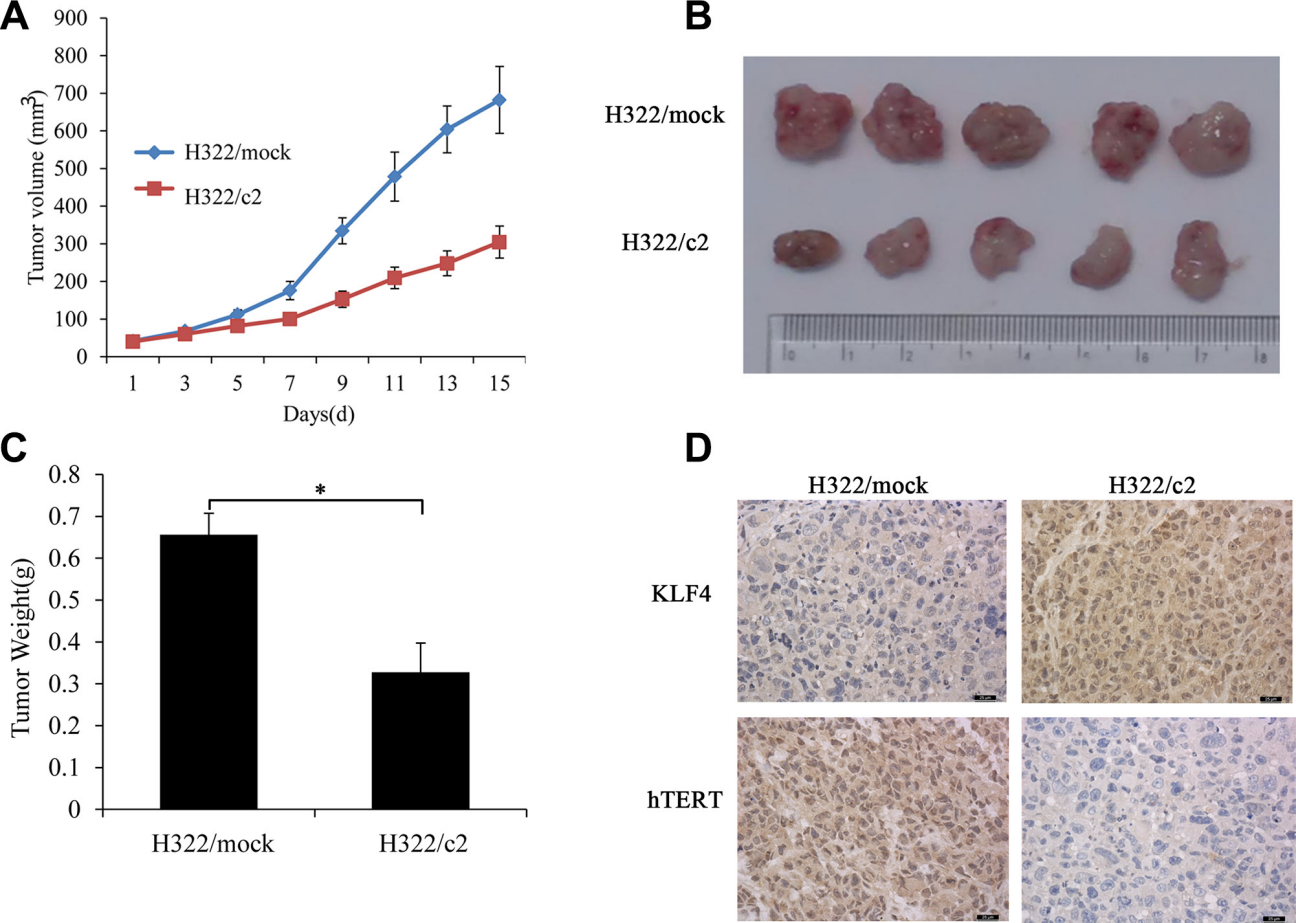
KLF4 suppresses lung tumor formation and hTERT expression *in vivo* (**A**) Tumor growth of KLF4-overexpressing H322 cells (H322/c2) and mock controls in nude mice. The tumor volume was calculated using the following equation: V = (width^2^ × length)/2. *P < 0.05. (**B**) Representative tumor grafts are shown, which were grown in nude mice injected with H322/mock and H322/c2 cells. (**C**) The tumor weight was compared between the H322/mock and H322/c2 groups. (**D**) Representative images of KLF4 and hTERT staining are presented in each group of nude mice using immunhistochemistry analysis.

On the basis of the above data, we next examined whether KLF4 also affected the tumor formation ability and hTERT expression *in vivo*. Xenograft assays were performed in nude mice, and the development and growth of solid tumors were monitored. We injected KLF4-overexpressing H322/c2 cells into nude mice, and H322/mock cells were used as the control. Consistent with its functional properties *in vitro*, mice infected with H322/c2 produced smaller (Figure 8A, 8B) and lighter (Figure 8C) tumors, and these tumors progressed much slower compared to the control. Next, we examined the expression of KLF4 and hTERT in xenograft tumor tissues using immunohistochemical staining. Loss of hTERT expression was observed in xenografts after KLF4 overexpression (Figure 8D). These results were consistent with the effect of altered KLF4 expression on cancer cell growth *in vitro* and hTERT expression as described above.

## DISCUSSION

In this study, using the combination of a lung tumor tissue microarray, cell lines and animal models, we investigated the interaction between KLF4 and hTERT in lung cancer. Initially, we performed an IHC analysis of TMA (tissue microarrays) containing normal lung and lung tumor tissues, which indicated that KLF4 negatively regulated hTERT expression. Survival analysis indicated that the combination of loss of KLF4 and the overexpression of hTERT were significantly correlated with poor prognosis in lung cancer patients. Consistent with these clinical observations, KLF4 overexpression resulted in decreased hTERT expression *in vitro* and *in vivo*, while the knockdown of KLF4 expression had the opposite effect. Further studies revealed that KLF4 was directly bound to the promoter regions of the hTERT gene, and the MAPK signaling pathway may participate in regulating cancer cell growth and hTERT expression. Furthermore, decreased KLF4 promoted lung cancer cell viability, adhesion, migration and cancer formation, while hTERT could partially rescue the suppressive function of KLF4 in cell proliferation. Conclusively, our findings provided clinical information and a potential mechanism supporting that KLF4 negatively regulate lung cancer cell growth and hTERT expression *in vivo* and *in vitro* via MAPK signal pathway, and the novel KLF4/hTERT/MAPK pathway may become a potential target for treating lung cancer in the future.

One of the emerging roles of KLF4 is the regulation of diverse tumor progression and its potential prognostic value for patient outcomes as it induces both malignant transformation and a slow-growth phenotype. This notion has been supported by a number of pieces of evidence. Numerous studies have indicated that KLF4 overexpression predicts a better prognosis in several malignancies, including prostate cancer, colon cancer, urothelial cancer and gastric cancer [14, 15, 40–41]. Lack of KLF4 and p21 expression has been associated with an accumulation of aggressive features in neuroendocrine lung tumors, and KLF4 is presented because of an aggressive phenotype in early-stage breast tumors [42, 43]. Consistent with these observations, our results revealed that KLF4 expression in the lung cancer tissues, compared with normal tissue, was lost. The combination of increased KLF4 expression and decreased hTERT expression were also associated with a longer relapse-free survival rate using Kaplan-Meier analysis, and the loss of KLF4 occurred in advanced lung tumors. Restoration of KLF4 expression in lung cancer cells inhibited cell growth *in vitro* and tumor formation *in vivo*. Moreover, forced restoration of hTERT expression rescued KLF4-mediated marked cell growth depression. Thus, KLF4 and hTERT can be used as novel biomarkers for predicting the outcomes of lung cancer patients. The clinical and experimental evidence strongly suggest that KLF4 functions as a tumor suppressor gene in human lung cancer and its altered expression plays an important role in lung cancer development and progression via regulating hTERT expression. KLF4 and hTERT expression can be used to design optimal individualized treatment as well as to distinguish between patients who would or would not benefit from close monitoring after surgery. Numbl, a polarity protein Numbl, was revealed to suppress the KLF4 ‘stemmness' transcriptaional program, resulting in shortened relapse-free survival in patients with lung cancer [44]. Taken together, restoration of KLF4 expression may produce potential in treating lung cancer in the future. KLF4, as biomarkers, it might be helpful tools for diagnostic, prognostic and monitoring purposes. Furthermore, these findings also support the perspective that potential therapeutic strategies based on telomerase, such as the telomerase inhibitor GRN163L and telomerase therapeutic vaccines, may offer new anticancer products and effective therapy strategies [26, 28, 45].

As an ambiguous cancer-related gene, KLF4 can function as an oncogene or tumor suppressor in specific cellular contexts, although the mechanisms underlying the contribution of KLF4 in malignancies have not been completely established [7, 46]. Previous studies have demonstrated that p21, a cell-cycle regulator, might function as a switch in converting KLF4 from a proliferation inhibitor into an oncogene [46]. Here, by directly binding to the promoter region of hTERT, KLF4 functions as a negative regulator of hTERT expression and telomerase activity *in vitro* and *in vivo*. Nevertheless, in a contradictory study, KLF4 is required to maintain hTERT expression and telomerase activity in hESC, FaDu (squamous cell carcinoma) and oral epidermoid carcinoma (OECM1) cell lines [47]. In ES cells, KLF4 promotes telomerase activity by recruiting β-catenin binding to the TERT gene promoter, which is contradictory to our results [32]. A potential explanation for this discrepancy may be that the regulation of hTERT by KLF4 exhibits tissue-specific characteristics, which may be from the different roles that KLF4 plays depending on the tumor type. Our study was conducted using lung cancer cell lines, while Chui-Wei Wong et al. performed overexpression studies in stem cells, squamous cell carcinoma cell lines and oral epidermoid carcinoma cell lines. This difference in the cell type is particularly significant with respect to the telomerase activity. These data may provide an explanation for the precise molecular basis of the differential roles that KLF4 plays in a variety of tumor types. Thus, more studies are needed to examine whether the difference in these two systems or genetic backgrounds contributes to the discrepancies observed between the previously reported results and our current results.

We further explored the molecular mechanisms of KLF4-mediated inhibition of hTERT expression. Principal molecular markers of the MAPK signaling pathway were tested in lung cancer cells that have altered KLF4 levels. Significant variations in KLF4 expression demonstrated that KLF4 might suppress lung cancer cell growth and hTERT expression via MAPK signaling. This hypothesis is supported by the following published literature. The ERK MAPK pathway is responsible for cell proliferation, differentiation and activation of this pathway is involved in tumor occurrence, invasion and metastasis [36, 48]. Overexpression and activation of MAPK pathway are commonly detected in colorectal cancer. Thus, new inhibitor of ERK MAPK could be a molecular target for colorectal cancer treatment [49]. In human non–small cell lung cancer, activated p38 was consistently increased in tumor compared with normal tissue, Therefore, activation of MAPK cascades may play a role in malignant cell growth or transformation [50] As a substantial growth-related signaling pathway, cells regulate telomerase activity via MAPK signaling in diverse cellular processes. Activation of the MAPK pathways contributes to the upregulation of telomerase activity in regenerating hepatocytes [38, 39]. Under hypoxic conditions, cancer cells promote telomerase activity via MAPK cascade signaling as a stress response [51]. In human endometrial cancer cells, estradiol-induced telomerase activity is mediated via the MAPK signaling pathway [37]. Moreover, hTERT has proved to be an identical target of Wnt/β-catenin signaling in cancer cells. Previous articles revealed that hTERT is a novel target of the Wnt/β-catenin pathway. Activation of the Wnt/β-catenin pathway elevated hTERT expression and telomerase activity. Furthermore, silencing endogenous β-catenin expression suppressed hTERT expression and telomerase activity effectively. In addition, KLF4 could directly interact with Wnt/β-catenin signaling to suppress Wnt signaling and inhibit tumor growth. Thus, Wnt/β-catenin signaling was demonstrated to play a role in the interaction between KLF4 and hTERT [30–32]. Further improved understanding of the mechanism underlying KLF4-mediated inhibition will result in novel therapies for treating lung cancer.

In summary, we identified the hTERT gene as a novel target of KLF4 in lung cancer and showed that KLF4 expression was negatively associated with hTERT in both patient tissue samples and lung cancer cell lines, demonstrating KLF4 plays a tumor suppressor role in lung tumor development and progression. Despite the limitations in our study, targeting the KLF4/hTERT/MAPK pathway may provide a new therapeutic strategy for controlling lung tumor progression.

## MATERIALS AND METHODS

### Human tissue specimens and immunohistochemical analysis

Ninety human lung cancer TMAs containing both human lung cancer tumor specimens and normal lung tissue specimens were used to analyze the KLF4 and hTERT expression levels. (Unfortunately, ten pairs were lost because of an improper experimental operation). The tissue microarrays were purchased from SOBC outdo Biotech, HLug-Ade180Sur. Slides were dried in an incubator at 65°C for 2 h. After routine treatment with xylene for deparaffinization, rehydration with ethanol and endogenous peroxidase activity blocking with 3% H_2_O_2_ in methanol, antigen retrieval was performed by 5 min of boiling using a pressure cooker in 0.1 mM sodium citrate buffer (pH 6.0). The section was blocked with 10% normal goat serum (C-0005, Bioss) in PBS for 30 min at room temperature and then incubated overnight at 4°C with primary anti-KLF4 (1:100; Santa Cruz Biotechnology, sc-20691) and anti-hTERT (1:50; Abgent, AP1410d) antibodies. Detection of the primary antibody and color development were performed using the GTvisionIII Immunohistochemical Assay Kit (HRP/DAB, rabbit/mouse-general, two-step) (GK500710, Gene Tech (Shanghai) according the manufacturer's protocol. Sections were subsequently counterstained with Mayer's hematoxylin, dehydrated and mounted onto coverslips. Images were acquired using polarized light microscopy. The stained tissue arrays were evaluated and scored based on the intensity and proportion. The staining intensity was graded as 0 (negative), 1 (weak), 2 (moderate), or 3 (strong), and the proportion was evaluated on a scale of 0 (0%), 1 (0.1%–1%), 2 (2%–10%), 3 (11%–33%), 4 (34%–66%), and 5 (67%–100%). The composite score of the intensity and proportion was used for statistical analysis.

### Cell lines and culture conditions

Human lung cancer cell lines (H322, H322/mock, H322/c2, H322/c5, H1299) were maintained in RPMI 1640 medium (HyCloneThermo scientific NYL1020) supplemented with 10% fetal bovine serum and 5% glutamine. Two stable KLF4-expressing clones of H322 cells (KLF4-c2 and KLF4-c5) were selected, and H322 cells transfected with empty vector were used as the control (H322/mock). A normal human fibroblast cell line, NHFB, was maintained in DMEM medium supplemented with 10% fetal bovine serum, and the A549 cell line was cultured in F12 medium supplemented with 10% fetal bovine serum. All of the cells were incubated in a humidified incubator supplied with 5% carbon dioxide.

### Transient transfection of lung cancer cells

Adenoviral KLF4 (Ad-KLF4) or adenoviral hTERT (Ad-hTERT) was transfected with cells to achieve overexpression of KLF4 or hTERT, respectively. LacZ, plasmid vector, was used as a nonspecific negative control. Retroviruses carrying small interfering RNA (siRNA) were used to knock down KLF4 or hTERT expression. KLF4 small-interfering RNA oligos (siRNA1: 5' GAGAGACCGAGGAGUUCAAdTdT 3' (sense) and 3' dTdTCUCUCUGGCUCCUCAAGUU 5' (anti-sense); siRNA2: 5' GCAGCUUCACCUAUCCGAUdTdT 3' (sense) and 3' dTdTCGUCGAAGUGGAUAGGCUA 5' (anti-sense); and siRNA3: 5' GACCAGGCACUACC GUAAAdTdT 3' (sense) and 3' dTdTCUGGUCCGUGAU GGCAUUU 5' (anti-sense)) and hTERT small-interfering RNA oligos (siRNA1: 5' GCUCGUGGAGACCAUC UUUdTdT 3' (sense) and 3' dTdTCGAGCACCUCUGGUA GAAA 5' (anti-sense); siRNA2: 5' GGCCGAUUGUG AACAUGGAdTdT 3' (sense) and 3' dTdTCCGGCUAAC ACUUGUACCU 5' (anti-sense); and siRNA3: 5' UGC GUUUGGUGGAUGAUUUdTdT 3' (sense) and 3' dTdT ACGCAAACCACCUACUAAA 5' (anti-sense)) were synthetically purchased from RIBBIO (Guangzhou) and used to knock down KLF4 or hTERT expression. Nontargeting siRNA (siCtrl) was used as the control. Cells were harvested at 72 hours after transfection and processed for subsequent experiments. All of the transfection experiments were performed using Sinofection^®^ Transfection Reagent (STF02; Sino Biological Inc.) according to the manufacturer's instructions.

### Western blotting analysis

Lung cancer cells were harvested 72 hours after transfection and solubilized in 200 μl of radio immunoprecipitation assay (RIPA) buffer supplemented with 1×PMSF. Next, 50 μg of whole-cell protein lysates were separated using electrophoresis on 10% to 12% SDS-PAGE, transferred ontopolyvinylidenedifluoride (PVDF) membranes (Millipore) and then incubated with primary antibodies, including KLF4 (1:500, Santa Cruz Biotech, sc-20691) and hTERT (1:1000, Abgent, AP1410d) and the secondary antibody (1:4000, Bioss, bs-0295G). Equal protein sample loading was monitored using an anti-GAPDH antibody (1:2000 Cell Signaling, #5174). Other primary antibody used consisted of p-erk (CST 4370), erk (CST, 4695), p-p38 (CST, 4511), p38 (CST, 8690), p-jnk (CST, 4668), jnk (CST, 9252). Reactive bands were visualized with ECL Plus reagents.

### RNA extraction and reverse transcription-polymerase chain reaction

Total RNA extraction was collected using the Trizol reagent (Invitrogen) according to the manufacturer's instructions. Next, RNA was phenol-chloroform-extracted, precipitated and dissolved in DEPC water. Next, 0.5 μg of total RNA was reverse transcribed to synthesize cDNA samples. Subsequently, 1 μl of cDNA products was subjected to polymerase chain reaction (PCR) amplification, and the following PCR primers were used:KLF4:5'-CGGACCACCTCGCCTTACA-3'(sense),5'-CTGGGCT CCTTCCCTCATCG-3' (anti-sense); hTERT 5' –CT GCTGCGCACGTGGGAAGCC-3' (sense), 5'-GTCC CCGCGCTGCACCAGCC-3' (anti-sense); glyceraldehyde 3-phosphate dehydrogenase 5' TGCACCACCAACT GCTTAG 3' (sense) 5' AGTAGAGGCAGGGATGA TGTTC 3' (anti-sense). The PCR products were loaded onto 1% agarose gels and visualized using ultraviolet light.

### Immunofluorescence

H322/mock, H322/c2 and H322/c5 cells were cultured in 1640 medium and plated in 6-well plates at a density of 1 × 10^5^ cells per well; then, 2–4 glass coverslips were placed in each of the wells. The plates were incubated for 24 to 48 hours until reaching 30–40% confluence prior to fixation; they were then fixed with 4% paraformaldehyde for 10 min and permeabilized with 0.1% Triton X-100 in PBS. The slides were washed three times with PBS and blocked with 5% BSA in PBS for 30 min at room temperature. The cells were incubated with the KLF4 antibody (1:100; Santa Cruz Biotechnology, sc-20691) or hTERT antibody (1:100; Santa Cruz Biotechnology; sc-68720) overnight at 4°C, which was followed by incubation with a fluorescein isothiocyanate (FITC)-conjugated anti-rabbit secondary antibody (1:100; Thermo Scientific) for 30 min in the dark. Nuclei were counterstained with DAPI for 15 min and then analyzed using a fluorescent microscope.

### Chromatin immunoprecipitation assay (ChIP)

ChIP assays were performed using reagents obtained from Cell Signaling Technology (SimpleChIP^®^ Enzymatic Chromatin IP Kit (Magnetic Beads) #9003) according to the manufacturer's instructions. Briefly, 4 × 10^7^ cells were fixed with 37% formaldehyde for 10 min at room temperature and were neutralized by the addition of glycine for 5 min at room temperature. The cells were then lysed and chromatin was harvested and fragmented using sonication and enzymatic digestion. The chromatin was then subjected to immunoprecipitation using an anti-KLF4 (Santa Cruz Biotech, sc-20691), anti-histone 3 and normal rabbit-IgG antibody. ChIP-enriched DNA was quantified using real-time PCR, and the primer sets were designed as follows: hTERT: 5'-CGGACCACCTCGCCTTACA-3'(sense) and 5'-CTGGGCTCCTTCCCTCATCG-3' (anti-sense). The PCR products were loaded onto 2% agarose gels and visualized with ultraviolet light.

### Streptavidin-agarose pulldown assay

The selected cells were harvested and processed to prepare nuclear extracts. Nuclear extract proteins (200 μg) obtained from cancer cells were incubated with 5' biotinylated double-stranded hTERT promoter probe (sense: 5' CTGCTGCGCACGTGGGAAGCC 3' and anti-sense: 5' GTCCCCGCGCTGCACCAGCC 3') and 30 μL of streptavidin-agarose beads. The mixture was placed on a rotating shaker with gentle mixing at 4°C overnight. After incubation, the tubes were centrifuged at 5000 g in a microcentrifuge for 30 s, and the supernatant was removed. The pellet was washed three times with ice-cold PBS. The pulled down mixture was resuspended in 30 μL of 4×loading buffer and heated for 5 min at 100°C. The proteins in the complex were dissociated and analyzed using Western blot analysis. Next, 20 μL of the KLF4 antibody (Santa Cruz Biotech, sc-20691) was used in the test, and a normal rabbit-IgG (1 μL/mL) was used as the control.

### Luciferase reporter assay

For the luciferase assays, H1299/hTERT-luc cells were plated at 5 × 10^5^ cells per 60-mm well and then infected with different doses of Ad/CMV-LacZ and Ad/CMV-KLF4. Forty-eight hours later, the cells were washed and harvested for the luciferase assay, which was followed by treatment with the Tropix Luciferase Assay kit (Tropix, Bedford, MA, USA). Bioluminescence was detected using a Monolight 2010 luminometer (BD Pharmingen, San Diego, CA, USA). For each plasmid construct, three independent transfection experiments were performed in triplicate. The structures of hTERT promoter-luciferase constructs were shown in Figure 5B, which were cloned previously [52].

### Telomerase activity measurement

Telomerase activity was assessed using the TeloTAGGG Telomerase PCR ELISA kit (Roche Applied Science, Indianapolis, IN) according to the manufacturer's instructions. Briefly, 2 × 10^5^ cells per sample were harvested and transferred; the pelleted cells were resuspended in 200 μL of lysis reagent and incubated on ice for 30 min. After centrifugation at 16,000 × g for 20 min at 4°C, the supernatants from the lysates were collected. The cell extracts (3 μL, corresponding to 3 × 10^3^ cell equivalents) were then incubated with biotinylated telomerase substrate oligonucleotide (P1-TS primer) at 25°C for 20 min. Telomerase inactivation was performed at 94°C for 5 min. The extended products were amplified by PCR using P1-TS and P2 primers for 30 cycles. Biotinylated telomeric repeat amplification products were incubated for 2 h at 37°C with a digoxigenin-labeled detection probe complementary to the telomeric repeat sequence and immobilized onto streptavidin-coated microtiter plates. Next, the wells were incubated for 30 min at room temperature with a peroxidase-labeled antidigoxigenin polyclonal antibody. Finally, the level of telomeric repeat amplification products was determined after the addition of the peroxidase substrate (3,3',5,5'-tetramethylbenzidine). The absorbance of each batch was measured at a wavelength of 450 nm (with a reference wavelength of 690 nm; Vmax kinetic microplate reader, Molecular Devices) within 30 min after the addition of the stop reagent. The 293 cell line was used as the positive control; heat-treated (85°C for 10 min) 293 cells served as the negative controls. In addition, the samples and controls were tested in the sample batch throughout the study. Each experiment was performed in quadruplicate on the same day and repeated three times.

### Cell proliferation assay

H1299 and A549 Cells were seeded onto a 96-well culture plate at a density of 2 × 10^4^ overnight, transfected with targeted siRNAs or controls, and cultured for 72 hours. Cell viability was evaluated using the MTS assay (CellTiter 961 AQueous One Solution Cell Proliferation Assay, Promega) according to the manufacturer's instructions. The absorbance was recorded at 490 nm using a BioTek ELx800 absorbance microplate reader. The GraphPad Prism Software version 6 for Windows was used.

### Cell migration assay

Next, 5 × 10^6^ cells were plated onto the prepared 60-mm dish and infected with the KLF4-specific siRNA or controls. The plate was then incubated overnight at 37°C, allowing the cells to adhere and completely spread onto the substrate. Wounds were made using a p100 pipette tip. The debris was removed and the cells were washed with 1 mL of PBS twice and replaced with 2 mL of complete medium. After the reference points were made, the scratch was observed and recorded under a phase-contrast microscope, and the first image of the scratch was acquired. Next, the dish was placed in a tissue culture incubator at 37°C for 24 h. The cells were imaged at 0 and 24 h and the gap widths were measured using Image J software. The experiment was repeated three times.

### Cell attachment assay

The treated cells (2 × 10^4^) were seeded onto 96-well cells plates for 6 hours. Next, the medium was discarded and the cells were washed twice with PBS. The attached cells were then fixed with 4% paraformaldehyde for 10 min, and subsequently stained with 0.2% crystal violet for 20 min. The stained cells were washed thoroughly and dissolved completely in 1% sodium dodecyl sulfate. The absorbance at 570 nm was measured.

### EdU incorporation assay

Keyfluor Click-iT EdU flow cytometry analysis was performed according to the manufacturer's instructions (KGA322-50, KeyGen BioTech). Briefly, cells infected with Ad-KLF4, Ad-vector or Ad-hTERT were incubated in 6-well culture dishes. After three days, 10 uL of EdU was added to the culture medium at the desired final concentration and thoroughly mixed, which was followed by a 6 h incubation. The cells were washed with 3 mL of 1% BSA in PBS and were pelleted by centrifugation; then, the supernatant was removed. The cells were fixed with 1 mL of 4% paraformaldehyde fixative for 15 minutes at room temperature while protected from light. The cell pellet was dislodged and resuspended in 100 μL of 1% sapon and permeabilized for 20 minutes. Next, 0.5 mL of the Click-iT Plus reaction cocktail was added into each tube and mixed well. The reaction mixture was incubated for 30 minutes at room temperature while protected from light. The samples were then analyzed using a flow cytometer.

### Tumor xenograft assay

All of the experiments involving animals were approved by the Institutional Animal Care and Use Committee. Tumor cells (H322/mock or H322/c2) were prepared and injected into the subcutaneous layer or pancreas of nude mice. All mice were sacrificed 25 or 45 days after tumor cell injection or when they appeared moribund. Tumor tissues were harvested, weighed, and processed for further analyses. The tumor tissues were formalin-fixed, paraffin-embedded, and analyzed using immunohistochemistry for KLF4 and hTERT expression. The antibodies used for the immunohistochemistry assay were the KLF4 antibody (1:500, Santa Cruz Biotech, sc-20691) and hTERT antibody (1:1000, Abgent, AP1410d).

### Statistical analysis

Statistical analysis was performed using GraphPad Prism 6. For comparison of paired tissues, the paired Student's *t*-test was performed to detect statistical significance. Chi-square tests were used to analyze the correlation between the KLF4 expression and clinicopathological features of lung carcinoma. The correlation between KLF4 and hTERT expression levels in human lung cancer tissues was also determined using Chi-square tests. Survival curves were constructed using the Kaplan-Meier method and were compared using the log-rank test. The value is presented as the mean ± S.D. A *p*-value of < 0.05 was considered statistically significant, which has been labeled with an asterisk in the figures, while two asterisks indicate a *p*-value < 0.01. Additionally, a data portal (cBioPortal for Cancer Genomics) was used to analyze KLF4 and its relevant factors in lung cancer. The database query was based on deregulation (mutant and altered expression) of the KLF4 expression in human lung cancer. Statistical significance was set at *P* < 0.05 (see Supplementary Data).

## CONCLUSIONS

Our results provide insight into the role played by directly KLF4 binding to the promoter of hTERT and its ability to suppress hTERT expression and telomere activity *in vitro* and *in vivo*. KLF4 expression in lung cancer cells inhibited cell growth, and the overexpression of hTERT neutralized the effect of KLF4. Clinical data revealed the prognostic significance of KLF4 and hTERT expression in lung cancer. Consequently, a better understanding of the mechanism for KLF4-mediated inhibition may result in new therapeutic perspectives.

## SUPPLEMENTARY MATERIAL FIGURE


