# Supplementary material for: KLF4 downregulates hTERT expression and telomerase activity to inhibit lung carcinoma growth
Source: Oncotarget. 2016 May 2;7(33):52870–87. doi: 10.18632/oncotarget.9141 (PMC5288155; doi:10.18632/oncotarget.9141)
Supplement: Supplementary file 1 [file oncotarget-07-52870-s001.pdf]

KLF4 downregulates hTERT expression and telomerase activity to inhibit lung carcinoma growth

Supplementary Materials

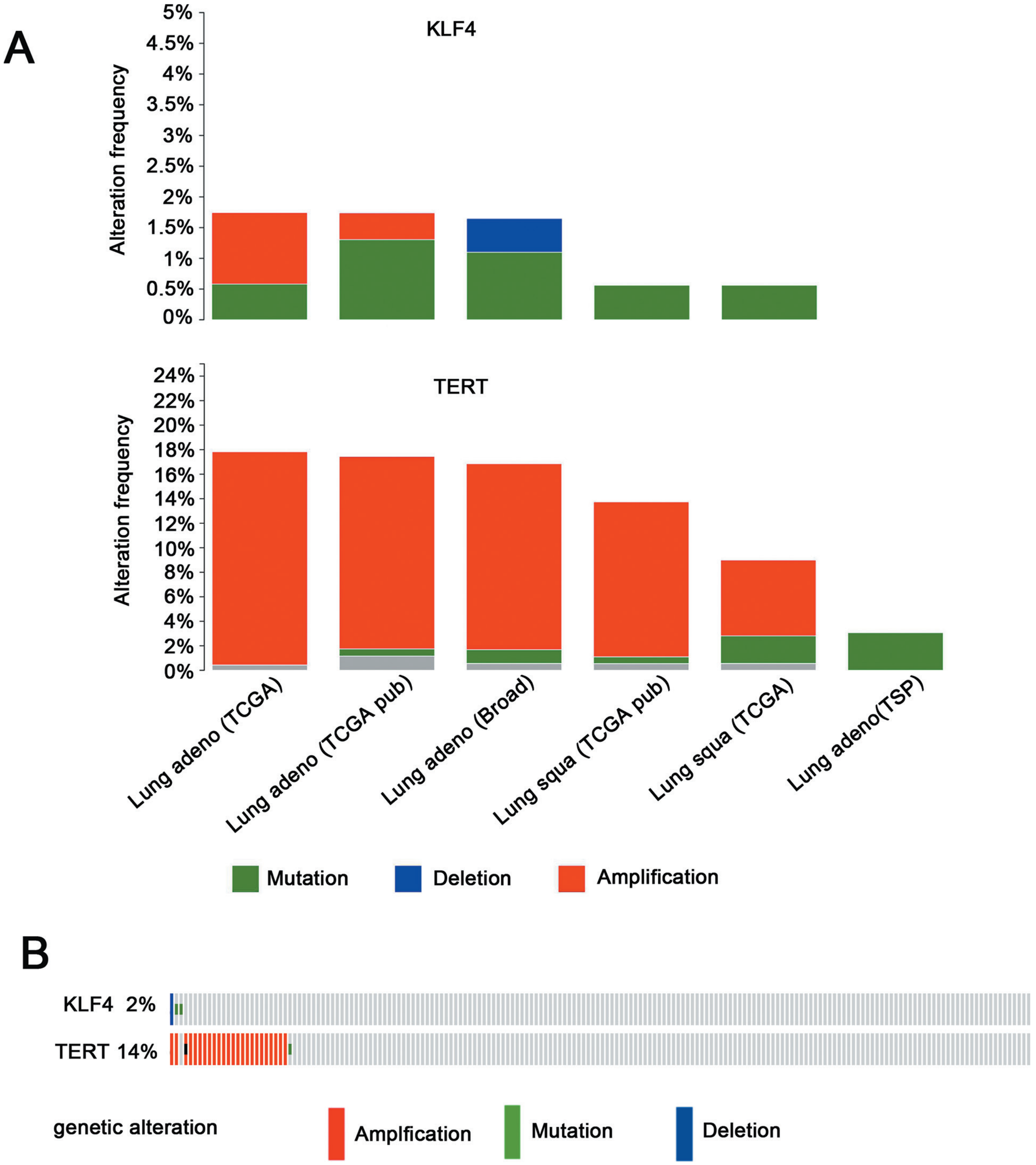

Supplementary Figure S1: Alternation of KLF4 and TERT expression in six lung carcinoma subsets (data from cBioPortal for cancer genomics: http://www.cbioportal.org/Gao et al. Sci. Signal. 2013 & Cerami et al. Cancer Discov. 2012). (**A**) KLF4 protein presented deletion or mutation (blue or green panels), whereas TERT indicated amplification in lung cancer (Red panel). (**B**) A mutual exclusive connection between KLF4 and TERT expression levels was presented in lung adenocarcinoma (TCGA, Nature 2014, 230 patients/230 samples) ($p < 0.05$).
